# Decrease in Admissions and Change in the Diagnostic Landscape in a Newborn Care Unit in Northern Ghana During the COVID-19 Pandemic

**DOI:** 10.3389/fped.2021.642508

**Published:** 2021-03-25

**Authors:** Alhassan Abdul-Mumin, Cesia Cotache-Condor, Kingsley Appiah Bimpong, Andie Grimm, Mary Joan Kpiniong, Rafiuk Cosmos Yakubu, Peter Gyamfi Kwarteng, Yaninga Halwani Fuseini, Emily R. Smith

**Affiliations:** ^1^Department of Pediatrics and Child Health, School of Medicine and Health Sciences, University for Development Studies, Tamale, Ghana; ^2^Department of Pediatrics and Child Health, Tamale Teaching Hospital, Tamale, Ghana; ^3^Department of Public Health, Robbins College of Health and Human Services, Baylor University, Waco, TX, United States; ^4^Duke Global Health Institute, Duke University, Durham, NC, United States

**Keywords:** COVID-19, neonatal mortality, pandemic impact, northern Ghana, newborn care

## Abstract

**Background:** The coronavirus disease (COVID-19) has spread worldwide with an increasing number of patients, including pregnant women and neonates. This study aims to evaluate morbidity and mortality in the COVID-19 era compared to the preceding year in the Neonatal Intensive Care Unit (NICU) at Tamale Teaching Hospital, Ghana.

**Methods:** This is a cross-sectional study carried out on neonates admitted to NICU between March 1st to August 31st, 2019 (pre-COVID-19 era) and March 1st to August 31st, 2020 (COVID-19 era). Multivariate logistic regression was performed to identify predictors of mortality for both periods.

**Results:** From 2,901 neonates, 1,616 (56%) were admitted before, and 1,285 (44%) were admitted during the pandemic. Admissions decreased during the COVID-19 era, reaching their lowest point between June and August 2020. Compared to the previous year, during the COVID-19 era, admissions of patients born at TTH, delivered at home, and with infections decreased from 50 to 39%, 7 to 4%, and 22 to 13%, respectively. Referred status (OR = 3.3) and vaginal delivery (OR = 1.6) were associated with an increased likelihood of mortality. For low- birth weight neonates, admissions of patients born at TTH, with vaginal and home delivery decreased from 62 to 48%, 8 to 2%, and 59 to 52%, respectively. Neonatal infections and congenital anomalies decreased from 8 to 4%, 5 to 3%, respectively. The likelihood of mortality among referred patients increased by 50%.

**Conclusion:** We observed a marked decrease in admissions and change in the diagnosis landscape and related mortality during the pandemic. Underlying challenges, including fear, financing, and health system capacity, might intensify delays and lack of access to newborn care in northern Ghana, leading to higher rates of lifelong disabilities and mortality. Immediate damage control measures, including an improved home-based continuum of care and equipping families to participate in the newborn care with complemented m-health approaches, are needed with urgency.

## Introduction

Severe acute respiratory syndrome-related coronavirus 2 (SARS-CoV-2) is the strain of the virus with the typical symptoms at the onset of illness, including fever, difficulty in breathing, dry cough, and fatigue (1). The disease caused by this virus was named Coronavirus disease 19 (COVID-19) by the WHO on February 11th, 2020 (2). On January 30th, 2020, the World Health Organization (WHO) declared COVID-19 a Public Health Emergency of International Concern and, subsequently, a pandemic on March 11th, 2020 (3, 4). Transmission of the disease is through human-to-human respiratory droplets from a cough or sneeze and fomites (5). Being exposed to the epidemic center and close contact with infected family members were identified as risk factors for transmission (6). COVID-19 has and is still spreading rapidly across the globe with an increasing number of patients, including pregnant women and neonates (7).

With no vaccines or antiviral treatments widely available for COVID-19, the pandemic's negative impacts are most present in overburdened healthcare systems (8). Studies have shown that disruptions in routine care plans may lead to a backlog of surgical cases that may take years to clear (9–11). Some studies have also suggested more indirect deaths could result from these disruptions caused by COVID-19 (10). There was a documented increase in indirect causes of maternal and neonatal mortality during the Ebola outbreak in Sierra Leone (12). Therefore, the fear is that the gains made in child mortality may be negatively affected by COVID-19, especially in countries with fewer resources. On March 12th, 2020, Ghana confirmed its first two cases of COVID-19. Since then, Ghana has had a rapid increase in the number of confirmed cases of the disease. Also, there have been reports of the temporary closure of some maternity units and Neonatal Intensive Care Units (NICUs) as a result of positive COVID-19 cases, with the country losing a pediatrician to the virus (13–15).

In recent times, children and pregnant women have become highly at risk due to their unique and specially modulated immune status (16, 17). It was found that the virus has potentially unfavorable outcomes for both mother and fetus during the perinatal period (18). Although reports have suggested a lower prevalence in pediatric populations (19–21), Castagnoli et al. (5), in their systematic review of 18 studies, identified 1,065 pediatric cases of SARS-CoV-2 infection. Clinical manifestations of the disease in children might be less severe as compared with adult patients (6, 7, 21–25), however, younger children and infants are more susceptible to infections (24). For example, a three-month-old has been among the COVID-19 deaths reported in Ghana (26).

Although the in-patient care and daily outpatient clinics for newborns continued during the peak of the restrictions (i.e., closure of out-patients services and cancellation of non-emergency surgeries) at Tamale Teaching Hospital (TTH) NICU in Northern Ghana, we are not sure if care-seeking in the catchment area might have been affected by external factors. Therefore, this study aims to describe the impact of the COVID-19 pandemic on newborn care by comparing morbidity and mortality between the COVID-19 era and the preceding year in the Neonatal Intensive Care Unit (NICU) at Tamale Teaching Hospital, Ghana.

## Methods

### Setting and Participants

The TTH is a tertiary facility that serves a 4-million catchment population and is the main center for medical students' clinical training from the University for Development Studies School of Medicine and Health Sciences. This facility receives patients from Northern, North East, Savannah, Upper East, and Upper West regions, sometimes from Burkina Faso, Cote d'Ivoire, and Togo. The hospital is equipped with a 50 incubator/crib capacity NICU with a 7-bed Kangaroo Mother Care Unit attached to it. Besides medical care, the unit also provides pre-operative and post-operative care for sick neonates referred from the catchment areas (27). The study included all neonates admitted to the NICU from March 1st to August 31st, 2019 (pre-COVID-19 era), and March 1st to August 31st, 2020 (COVID-19 era). Neonates were recruited for the study if they were admitted from TTH maternity unit or theater, from other facilities in the catchment area, or from home, and they aged up to 28 days at presentation. Neonates with significantly missing data, whose records were not entered into the electronic database or who were referred to other facilities were not included in the study.

### Study Design and Data Collection

This is a cross-sectional hospital-based study conducted at TTH NICU in northern Ghana to compare morbidity and mortality of neonatal admissions during the COVID-19 era to the period immediately preceding the pandemic. A local team of healthcare professionals at TTH (KAB and MJK) collected data from the TTH NICU between July and September 2020. AAM oversaw the data collection. The team used a predesigned data collection sheet to retrieve information on neonatal admissions, demographics, diagnosis, and outcomes from electronic records stored in the TTH NICU. For patients admitted two or more times, readmissions were not counted as new data entries. Instead, the first admission was included in this study. All information was deidentified and organized in an excel spreadsheet. COVID-19 incidence data (March-August 2020) were obtained from *Our World in Data*, a global database supported by the University of Oxford and Global Change Data Lab (28).

### Data Analysis

The pre-COVID-19 era was defined as neonatal admissions from March 1st to August 31st, 2019. The COVID-19 era was defined as neonatal admissions from March 1st to August 31st, 2020. Birth weight was defined as normal birth weight (NBW ≥ 2,500 grams) and low birth weight (LBW < 2,500 grams) to facilitate subset analysis and interpretation of policy implications (29). Descriptive and inferential statistics were generated using SAS 9.4 (SAS Institute Inc., Cary, North Carolina, USA). Demographic characteristics were compared among neonates admitted during the pre-COVID-19 era and the COVID-19 era using independent samples *t*-test and χ^2^ test when appropriate. Predictive variables included sex, region, referral, place of delivery, mode of delivery, birth weight, diagnosis, age of admission, length of stay, and insurance. The outcome was determined as a dichotomous variable (death/discharge). Bivariate and multivariate logistic regression analyses were performed to determine predictors of neonatal death. Only variables with *p* < 0.2 were included in the multivariate logistic regression model. Bivariate and multivariate logistic regressions were performed for neonates admitted during the pre-COVID-19 era and the COVID-19 era and in a subset population of neonates with birth weight <2500 g admitted during the pre-COVID-19 era and COVID-19 era. Statistical significance was defined at *p* < 0.05.

### Ethics Statement

Permission and ethical clearance to conduct this retrospective chart review was granted by the Research and Development Department and the Ethical Review Committee of the TTH, respectively.

## Results

**Figure 1:**
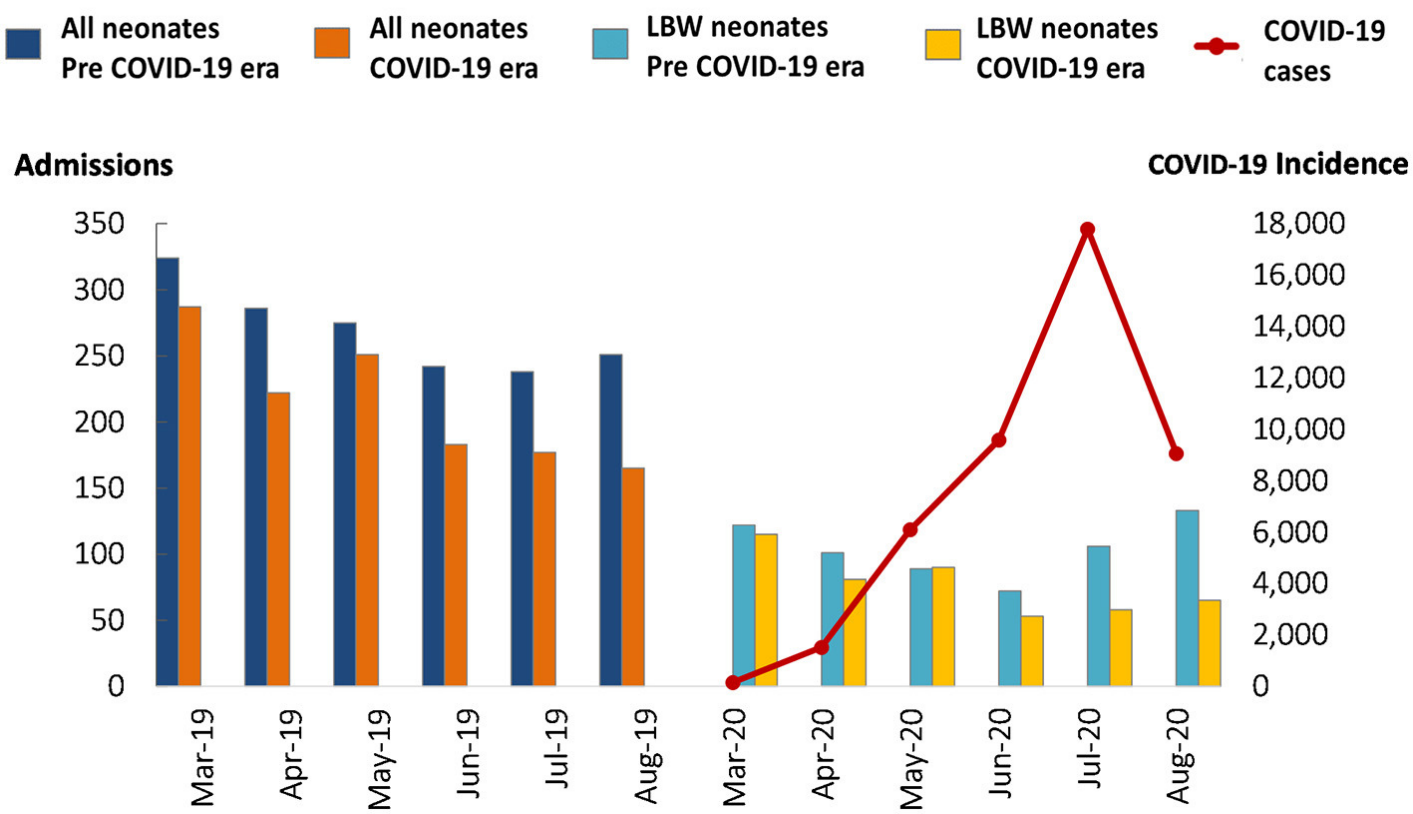
Frequency of neonates admitted in TTH NICU during pre-COVID-19 and COVID-19 era. Pre-COVID-19 was defined as neonatal admissions from March 1st to August 31st, 2019. COVID-19 era was defined as neonatal admissions from March 1st to August 31st, 2020.

**Figure 2:**
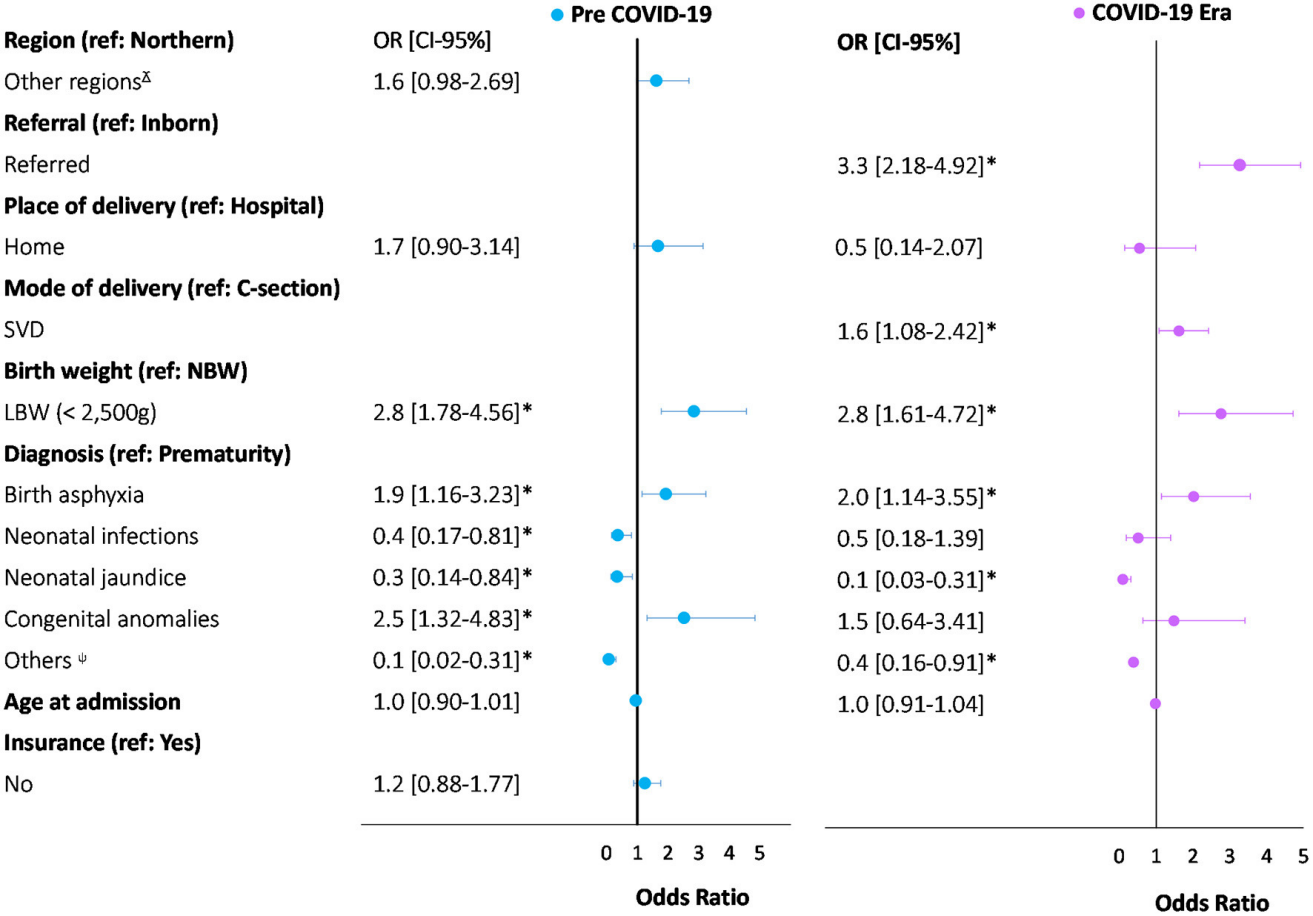
Comparison of multivariate logistic regression for predictors of neonatal death between pre-COVID-19 and COVID-19 era in TTH NICU. *Statistiscally significate (*p* < 0.05). ^ɤ^Includes: Ashanti, Bono, Bono East, North East, Oti, Savannah, Upper East, Upper West. ^ψ^Includes: pemphigous, dehydration, meconium aspiration syndrome, respiratory distress syndrome, retro-exposed, malnutrition, normal baby, stable baby, birth injuries, hematological diseases, neonatal seizures, low birth weight, and macrosomia. Pre-COVID-19 was defined as neonatal admissions from March 1st to August 31st, 2019. COVID-19 era was defined as neonatal admissions from March 1st to August 31st, 2020.

A total of 2,901 neonates were admitted to TTH NICU between March 1st to August 31st in 2019 and 2020. Of these admissions, 1,616 (55.7%) were admitted before, and 1,285 (44.3%) were admitted after the declaration of the pandemic. General and low birthweight admissions decreased during the COVID-19 era and reached their lowest point between June and August 2020, while national COVID-19 incidence increased exponentially since March 2020 (Figure 1). During the COVID-19 era, admissions of inborn neonates decreased from 50% (*n* = 797/1,616) to 39% (*n* = 501/1,285) and neonates born at home decreased from 7% (*n* = 105/1,616 to 4% (*n* = 47/1,285). However, there was an increase in the proportion of referrals to the NICU from other facilities from 50% (*n* = 798/1,616) to 61% (*n* = 775/1,285) (Table 1). Furthermore, admissions due to neonatal infections decreased from 22% (*n* = 352/1,616) to 13% (*n* = 170/1,285). In contrast, admissions due to prematurity and complications, and neonatal jaundice increased from 25% (*n* = 398/1,616) to 28% (*n* = 355/1,285) and from 14% (*n* = 227/1,616) to 18% (*n* = 233/1,285), respectively. Age at discharge (11 vs. 9.7 days) decreased by one day, on average. The predictors associated with neonatal mortality pre-COVID-19 era were LBW (OR = 2.8), congenital anomalies (OR = 2.5), and birth asphyxia (OR = 1.9). In contrast, the predictors associated with neonatal mortality during the COVID-19 era were being referred from other facilities (OR = 3.3), LBW (OR = 2.8), birth asphyxia (OR = 2.0), and SVD (OR = 1.6) (Figure 2).

**Table 1:** General characteristics of neonates admitted in TTH NICU during pre-COVID-19 and COVID-19 era.

**Variable**	**TOTAL**		**PRE-COVID-19**		**COVID-19 ERA**		***p***
	***% (n)***		***% (n)***		***% (n)***		
	***mean ± SE***		***mean ± SE***		***mean ± SE***		
	**100.0 (2,901)**		**55.7 (1,616)**		**44.3 (1,285)**		
**Sex**							
Female	43.8	(1,270)	43.8	(708)	43.7	(562)	0.9555
Male	56.2	(1,630)	56.2	(907)	56.3	(723)	
Unknown	(1)						
**Region**							
Northern region	90.2	(2,472)	91.0	(1,354)	89.3	(1,118)	0.1362
Other regions^ɤ^	9.8	(268)	9.0	(134)	10.7	(134)	
Unknown	(161)						
**Referral**							
Inborn	45.2	(1,298)	50.0	(797)	39.3	(501)	<0.0001
Referred	54.8	(1,537)	50.0	(798)	60.7	(775)	
Unknown	(30)						
**Place of delivery**							
Home	5.9	(152)	7.1	(105)	4.3	(47)	0.0022
Hospital	94.1	(2,423)	92.9	(1,367)	95.7	(1,056)	
Unknown	(326)						
**Mode of delivery**							
Spontaneous vaginal delivery	62.6	(1,811)	63.7	(1,026)	61.1	(785)	0.1526
C-section	37.4	(1,083)	36.3	(584)	38.9	(499)	
Unknown	(7)						
**Birth Weight (g)**							
NBW (≥2,500 g)	60.2	(1,640)	61.0	(973)	59.1	(667)	0.3218
LBW (<2,500 g)	39.8	(1,085)	39.0	(623)	40.9	(462)	
Unknown	(176)						
**Diagnosis**							
Prematurity and complications	26.0	(753)	24.6	(398)	27.6	(355)	<0.0001
Birth asphyxia	20.1	(583)	19.7	(318)	20.6	(265)	
Neonatal infections	18.0	(522)	21.8	(352)	13.3	(170)	
Neonatal jaundice	15.9	(460)	14.0	(227)	18.1	(233)	
Congenital anomalies	6.8	(197)	7.1	(114)	6.5	(83)	
Others^ψ^	13.3	(386)	12.8	(207)	13.9	(179)	
**Insurance**							
No	33.6	(861)	34.0	(549)	33.0	(312)	0.6209
Yes	66.3	(1,700)	66.0	(1,067)	67.0	(633)	
Unknown	(340)						
**Outcome**							
Discharge	84.7	(2,457)	85.8	(1,386)	83.4	(1,071)	0.0720
Death	15.3	(444)	14.2	(230)	16.6	(214)	
**Age at admission (days)**	3.9 ± 0.1		4.1 ± 0.1		3.6 ± 0.1		0.0133
**Age at discharge (*****n*** **= 4015, days)**	10.5 ± 0.2		11.0 ± 0.2		9.7 ± 0.2		<.0001
**Age at death (*****n*** **= 752, days)**	7.8 ± 0.4		8.2 ± 0.6		7.4 ± 0.5		0.2457
**Length of stay (days)**	6.1 ± 0.1		6.5 ± 0.2		5.7 ± 0.1		0.0010

*Analysis includes independent samples t-test and χ^2^ test statistics when appropriate*.

ɤ *Includes: Ashanti, Bono, Bono East, North East, Oti, Savannah, Upper East, Upper West*.

ψ *Includes: pemphigous, dehydration, meconium aspiration syndrome, respiratory distress syndrome, retro-exposed, malnutrition, normal baby, stable baby, birth injuries, hematological diseases, neonatal seizures, low birth weight, and macrosomia*.

*Pre-COVID-19 was defined as neonatal admissions from March 1st to August 31st, 2019. COVID-19 era was defined as neonatal admissions from March 1st to August 31st, 2020*.

**Figure 3:**
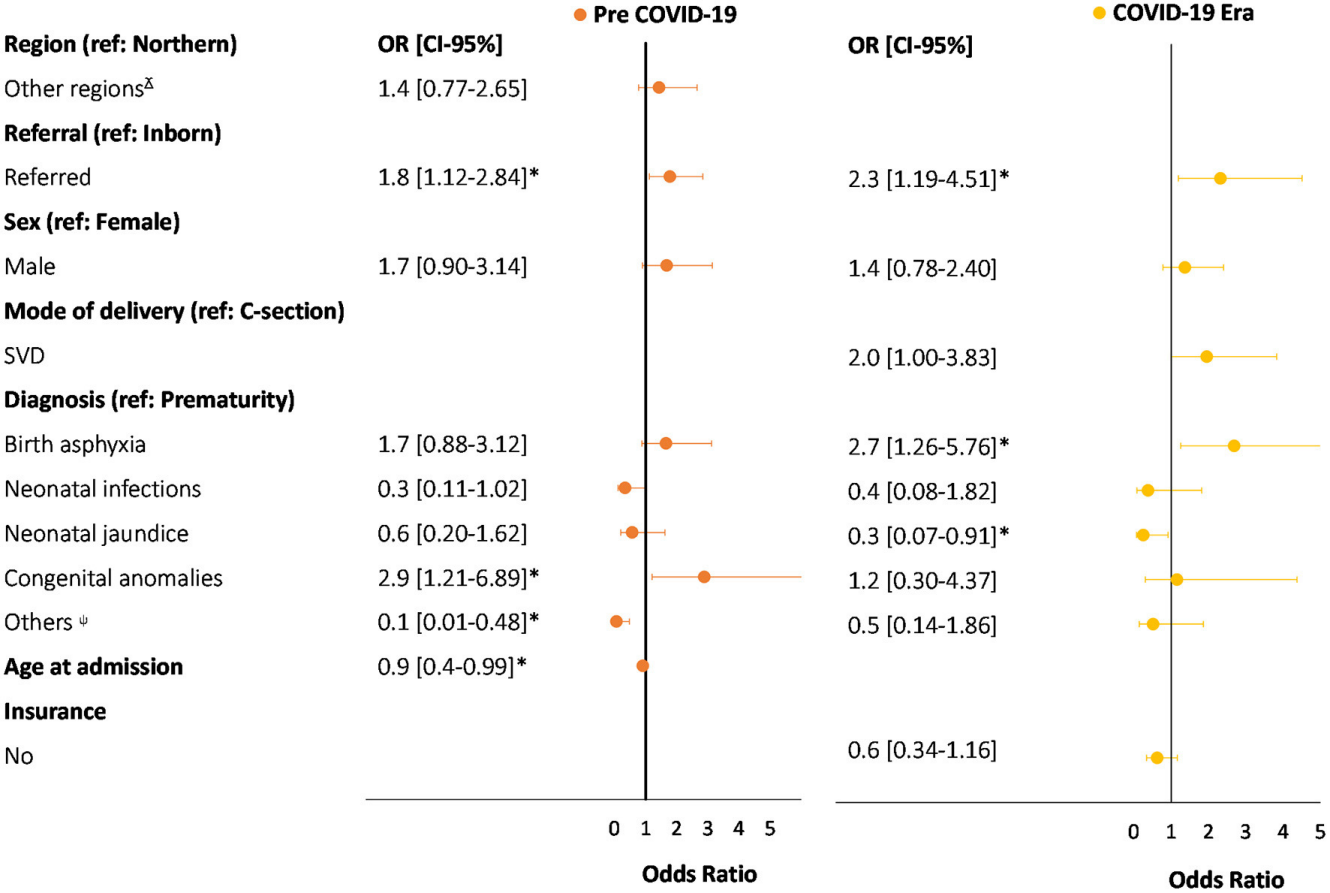
Comparison of multivariate logistic regression for predictors of neonatal death between pre-COVID-19 and COVID-19 era in LBW neonates (<2,500 g) in TTH NICU. *Statistiscally significate (*p* < 0.05). ^ɤ^Includes: Ashanti, Bono, Bono East, North East, Oti, Savannah, Upper East, Upper West. ^ψ^Includes: pemphigous, dehydration, meconium aspiration syndrome, respiratory distress syndrome, retro-exposed, malnutrition, normal baby, stable baby, birth injuries, hematological diseases, neonatal seizures, low birth weight, and macrosomia. Pre-COVID-19 was defined as neonatal admissions from March 1st to August 31st, 2019. COVID-19 era was defined as neonatal admissions from March 1st to August 31st, 2020.

When stratified by low birth weight (<2,500 grams), admissions of inborn neonates decreased from 62% (*n* = 386/623) to 48% (*n* = 220/462), neonates born at home decreased from 8% (*n* = 48/623) to 2% (*n* = 10/462), and neonates born by SVD decreased from 59% (*n* = 368/623) to 52% (*n* = 241/462). However, there was an increase in the proportion of referrals to the NICU from other facilities from 38% (*n* = 235/623) to 52% (*n* = 240/462), and neonates born by caesarian section increased from 41% (*n* = 254/623) to 48% (*n* = 221/462) (Table 2). Furthermore, admissions due to neonatal infections and congenital anomalies decreased from 8% (*n* = 53/623) to 4% (*n* = 17/462) and from 5% (*n* = 31/623) to 3% (*n* = 15/462), respectively. In contrast, admissions due to prematurity and complications, and neonatal jaundice increased from 60% (*n* = 377/623) to 66% (*n* = 307/462) and from 6% (*n* = 39/623) to 9% (*n* = 40/462), respectively. Age at discharge (12.7 vs. 10 days) and length of stay (9.4 vs. 7 days) decreased by two days, on average. The predictors associated with neonatal mortality pre-COVID-19 era were congenital anomalies (OR = 2.9) and being referred from other facilities (OR = 1.8). In contrast, the predictors associated with neonatal mortality during the COVID-19 era were birth asphyxia (OR = 2.7) and being referred from other facilities (OR = 2.3). The likelihood of mortality during the COVID-19 era compared to the pre-COVID-19 era increased by 50% among referred neonates (2.3 OR vs. 1.8 OR) (Figure 3).

**Table 2:** General characteristics of LBW neonates (< 2500 g) admitted at TTH NICU during pre-COVID-19 and COVID-19 era.

**Variable**	**TOTAL**		**PRE-COVID-19**		**COVID-19 ERA**		***p***
	***% (n)***		***% (n)***		***% (n)***		
	***mean ± SE***		***mean ± SE***		***mean ± SE***		
	**100.0 (1,085)**		**57.4 (623)**		**42.6 (462)**		
**Sex**							
Female	48.0	(521)	46.5	(290)	50.0	(231)	0.2606
Male	52.0	(564)	53.5	(333)	50.0	(231)	
**Region**							
Northern region	88.6	(903)	88.7	(501)	88.5	(402)	0.9497
Other regions^ɤ^	11.4	(116)	11.3	(64)	11.5	(52)	
Unknown	(66)						
**Referral**							
Inborn	56.1	(606)	62.2	(386)	47.8	(220)	<0.0001
Referred	43.9	(475)	37.8	(235)	52.2	(240)	
Unknown	(4)						
**Place of delivery**							
Home	5.7	(58)	8.1	(48)	2.3	(10)	<0.0001
Hospital	94.3	(957)	91.9	(542)	97.7	(415)	
Unknown	(70)						
**Mode of delivery**							
Spontaneous vaginal delivery	56.2	(609)	59.2	(368)	52.2	(241)	0.0216
C-Section	43.8	(475)	40.8	(254)	47.8	(221)	
Unknown	(1)						
**Diagnosis**							
Prematurity and complications	63.0	(684)	60.5	(377)	66.4	(307)	0.0038
Birth asphyxia	9.9	(107)	9.5	(59)	10.4	(48)	
Neonatal infections	6.5	(70)	8.5	(53)	3.7	(17)	
Neonatal jaundice	7.3	(79)	6.3	(39)	8.7	(40)	
Congenital anomalies	4.2	(46)	5.0	(31)	3.2	(15)	
Others^ψ^	9.1	(99)	10.2	(64)	7.6	(35)	
**Outcome**							
Discharge	77.5	(841)	77.8	(485)	77.1	(356)	0.7571
Death	22.5	(244)	22.2	(138)	22.9	(106)	
**Insurance**							
No	37.1	(357)	38.8	(242)	33.9	(115)	0.1312
Yes	62.9	(605)	61.2	(381)	66.1	(224)	
Unknown	(123)						
**Age at admission (days)**	2.4 ± 0.1		2.4 ± 0.1		2.2 ± 0.2		0.4138
**Age at discharge (*****n*** **= 841, days)**	11.5 ± 0.3		12.7 ± 0.5		10.0 ± 0.4		<0.0001
**Age at death (*****n*** **= 244, days)**	8.1 ± 0.5		8.8 ± 0.8		7.1 ± 0.6		0.0753
**Length of stay (days)**	8.4 ± 0.3		9.4 ± 0.4		7.0 ± 0.3		<0.0001

*Analysis includes independent samples t-test and χ^2^ test statistics when appropriate*.

ɤ *Includes: Ashanti, Bono, Bono East, North East, Oti, Savannah, Upper East, Upper West*.

ψ *Includes: pemphigous, dehydration, meconium aspiration syndrome, respiratory distress syndrome, retro-exposed, malnutrition, normal baby, stable baby, birth injuries, hematological diseases, neonatal seizures, low birth weight, and macrosomia*.

*Pre-COVID-19 was defined as neonatal admissions from March 1st to August 31st, 2019. COVID-19 era was defined as neonatal admissions from March 1st to August 31st, 2020*.

## Discussion

In this cross-sectional hospital-based study, we sought to document the early impact of COVID-19 on neonatal admissions and deaths by comparing data in our unit between the COVID-19 era and the pre-COVID-19 era. Overall, we found a significant decrease in neonatal admissions and changes in the landscape of diagnosis and their associations with mortality during the pandemic. These changes and additional challenges, including limited healthcare capacity, financial constraints, and widespread fear, might severely undermine newborn care in northern Ghana.

The Northern region is one of the poorest regions in Ghana (30). With the advent of the COVID-19 pandemic, the disparities in access to newborn care in this region have intensified. Our data suggest a decrease in admission from neonates born at home during the COVID-19 era. As part of the pandemic response and “the stay at home unless necessary” slogan, patients and their families were advised to refrain from visiting health facilities unless essential (31). In northern Ghana, where healthcare-seeking behaviors are already sub-optimal, the implementation of strict protocols of decongestion at TTH might have contributed to the step back from the most vulnerable communities' progress. Previous studies have found about 40% of women in northern Ghana deliver at home (32). For women who prefer to give birth at home due to traditional beliefs, lack of trust in the healthcare system, and financial challenges (33–35), additional challenges such as lockdown measures and fear of infection may have a significant influence when deciding to seek care at a health facility (36, 37). Previous literature has reported an 8% decrease in deliveries at health facilities during other health crises (38). If that is true for COVID-19 in northern Ghana, then the proportion of newborns without access to health care increases, thereby escalating the number of preventable neonatal deaths.

Decreased healthcare utilization is potentially explained by the fear of the virus and the lack of financial resources. Previous studies on knowledge, attitudes, and practices toward the pandemic in low-resource settings have shown disparities in understanding and performing the best preventative measures (39). This, in turn, can affect caregivers' decision-making when seeking medical and surgical care for their children amid a global pandemic. A previous study reported a decline in visits to pediatric emergency departments due to fear of contracting the coronavirus infection in Italy (40). Healthcare utilization is often negatively impacted by limited financial and medical resources. On average, for neonatal treatment of perinatal asphyxia and low birthweight in Ghana, out-of-pocket costs for care account for 66% of costs, and families often spend 9% of their annual income on acute care (41). Moreover, we have already seen increased closures of maternity units and NICUs within Ghana (13, 14). These shifts in resources and personnel often negatively impact emergency and essential surgical care, such as Cesarean sections (42). Such reductions in maternal and child health provisions have been estimated to result in 28,000 additional maternal deaths and 168,000 additional newborn deaths in LMICs (43).

We documented an increase in referrals and related likelihood of neonatal mortality during the COVID-19 era, compared to the pre-pandemic era. The increase in referrals could be explained by the fact that most lower health facilities were not operating at full capacity, and some facilities suspended operations temporarily due to exposure of staff to the coronavirus infection. In addition, antenatal clinics were not functioning at full capacity, with a resultant decrease in pregnancy monitoring. This might explain the increased likelihood of mortality among referred babies, as our facility received most of the complicated pregnancies and critically ill neonates from the catchment area. In the Ebola epidemic in Sierra Leone, a decline in utilization of vital reproductive, maternal and neonatal health services was reported, with about 22% decrease in antenatal care coverage and a consequent increase in neonatal deaths (38). Furthermore, SVD delivery was associated with an increase in neonatal mortality. This could be explained by the late presentation of pregnant women to the hospital due to initial hesitancy, leading to complicated deliveries (37).

Another collateral effect of the COVID-19 pandemic in TTH NICU is the drastic change of the diagnostic landscape and associated likelihood of mortality. We observed an increase in prematurity and complications, and neonatal jaundice admissions but a decrease in admissions from neonatal infections and congenital anomalies. The increase in jaundice cases could be explained by the early discharge of mother-infant pairs from the hospital, and the fewer opportunities for follow-up at postnatal clinics as most of these clinics suspended services during the peak of the pandemic. This potentially increased the risk for severe neonatal jaundice, a significant contributor of neonatal mortality and long-term disability related to neurocognitive issues, cerebral palsy, deafness, and learning difficulties (44–46).

Neonatal infections, birth asphyxia, and congenital anomalies remain among the leading causes of morbidity and mortality among neonates in LMICs (47–50). However, our results show the profile of neonatal admissions in the COVID-19 era look different from the pre-COVID-19 era, with fewer admissions related to neonatal infections and congenital anomalies, increased likelihood of mortality due to birth asphyxia (especially for LBW neonates), and decreased likelihood of mortality due to congenital anomalies. In normal times, admissions to our NICU due to birth asphyxia and other peripartum adverse events occur on the day of birth and from the delivery rooms, while admissions due to infections and other conditions occur later in the first week and from home. The decrease in admissions of neonatal infections during the pandemic could be explained by the increase in non-presentation—as these patients usually present from home—and/or the increased measures in hand hygiene and use of personal protective equipment during the peak of the pandemic in Ghana. On the other hand, the likelihood of mortality due to birth asphyxia could have been intensified by the decrease in admissions related to other diagnoses.

Congenital anomalies disproportionately affect children in LMICs and, if left untreated, can result in lifelong disabilities and death (51, 52). Before the pandemic, patients with congenital anomalies usually presented to TTH from home. Our data suggest that admissions and likelihood of mortality for these patients decreased during the COVID-19 pandemic. As previously discussed, the decrease in admissions is most likely explained by the widespread fear among caregivers and the preexisting, if not intensified, financial barriers in low resource settings. In consequence, congenital anomalies were not seen as a leading predictor of death during the COVID-19 pandemic, as it was pre-pandemic. The surgical backlog generated by underreported congenital anomalies contributes to the hidden neonatal mortality and the underestimation of the burden of disease in northern Ghana.

### Recommendations

Communities should be empowered with accurate information regarding the prevention and treatment of SARS-CoV-2 to increase healthcare utilization for the most vulnerable populations, such as pregnant women and sick newborns (42). Immediate damage control measures, including an improved home-based continuum of care and equipping families to participate in the newborn care with complemented m-health approaches are needed to mitigate the impact of the COVID-19 pandemic in newborn care in northern Ghana (31). As we battle with the second wave of the pandemic, effective care for neonatal jaundice should include risk factor screening, the early establishment of breastfeeding, monitoring and managing hyperbilirubinemia, and parental education before neonate discharge (53). To continue to prepare hospitals for the direct and indirect impacts of the pandemic, resources for surgical care should be improved, health care personnel should be educated on available treatments for SARS-CoV-2, and hospitals should ensure the provision of personal protective equipment (42, 54).

### Limitations

Our study constitutes a foundational step in identifying neonatal care challenges in northern Ghana amidst the COVID-19 crisis. However, some limitations warrant discussion. First, our study shares the limitations of cross-sectional designs, such as the inability to make causal inferences and establish temporal relationships between exposures and outcome. However, we offer a baseline knowledge that might be useful for future studies with more robust designs. Second, this a hospital-based study, and its results are limited to be generalized to the whole population. Therefore, a collection of robust population-based data is needed to evaluate newborn care access in northern Ghana. Third, data on COVID-19 incidence was not found for the Northern region, specifically. Instead, we used incidence data at a national level. Third, this study does not include specific data that can accurately explain the changes found in the neonatal care at TTH during the COVID era. Further studies may focus on hospital capacity, healthcare personnel, and parental perspectives to obtain a comprehensive view of the barriers hindering neonatal care amid the pandemic. Future studies may also focus on implementing and evaluating interventions to decrease the adverse effects of COVID-19 in newborn care and guide the creation of a robust evidence-based emergency preparedness protocol for neonatal care in Ghana.

## Conclusion

We have documented a decline in neonatal admissions and change in the diagnosis landscape and related mortality during the height of the COVID-19 restrictions in Ghana. As we battle with the second wave of the COVID-19 pandemic, common underlying factors such as widespread fear, financial challenges, and limitations in health system capacity might intensify direct and indirect impacts and contribute to more acute delays and lack of access to newborn care in northern Ghana. Consequently, higher rates of lifelong disabilities and preventable neonatal deaths due to leading causes of neonatal mortality and surgical backlog might be experienced in this country. Immediate damage control measures, including an improved home-based continuum of care and equipping families to participate in newborn care with complemented m-health approaches, are needed to mitigate the effects of the COVID-19 pandemic in newborn care in northern Ghana.

## Data Availability Statement

The raw data supporting the conclusions of this article will be made available by the authors, without undue reservation.

## Ethics Statement

The studies involving human participants were reviewed and approved by the Ethical Review Committee of the Tamale Teaching Hospital. Written informed consent from the participants' legal guardian/next of kin was not required to participate in this study in accordance with the national legislation and the institutional requirements.

## Author Contributions

AA-M conceived the study, sought relevant permissions for the study, supervised the data collection, provided guidance for the data analysis, and contributed to the initial drafts and revision of the manuscript. ES oversaw the project, provided guidance for the data analysis, and contributed to the study design and revision of the manuscript. KB and MK collected and cleaned the initial data and contributed to the initial draft and revision of the manuscript. CC-C analyzed the data, contributed to the study design, drafting, and revision of the manuscript. AG contributed to the initial drafting and revision of the manuscript. RY, PK, and YF supervised the data clean-up, contributed to the drafting and revision of the manuscript. All authors read and approved the final draft of the manuscript.

## Conflict of Interest

The authors declare that the research was conducted in the absence of any commercial or financial relationships that could be construed as a potential conflict of interest.
